# Decompressive craniectomy: Comparative analysis between surgical time and better prognosis

**DOI:** 10.3389/fneur.2022.1041947

**Published:** 2022-12-15

**Authors:** Luiz Severo Bem Junior, Ana Cristina Veiga Silva, Marcelo Diniz de Menezes, Maria Júlia Tabosa de Carvalho Galvão, Otávio da Cunha Ferreira Neto, Joaquim Fechine de Alencar Neto, Nicollas Nunes Rabelo, Nivaldo Sena Almeida, Marcelo Moraes Valença, Hildo Rocha Cirne de Azevedo Filho

**Affiliations:** ^1^Department of Neurosurgery, Hospital da Restauração, Recife, Brazil; ^2^Neuroscience Post-Graduate Program, Federal University of Pernambuco, Recife, Brazil; ^3^College of Medical Sciences, Unifacisa University Center, Campina Grande, Brazil; ^4^College of Medical Sciences, Pernambucana College of Healthy, Recife, Brazil; ^5^College of Medical Sciences, Federal University of Pernambuco, Recife, Brazil; ^6^College of Medical Sciences, Catholic University of Pernambuco, Recife, Brazil; ^7^Department of Neurosurgery, University of São Paulo, São Paulo, Brazil

**Keywords:** decompressive craniectomy, hemicraniectomy, malignant ischemic infarction, stroke, ischemic stroke

## Abstract

**Background:**

Malignant ischemic stroke is characterized by the involvement of 2/3 of the area of the middle cerebral artery, associated with cerebral edema, intracranial hypertension (ICH) and cerebral herniation, generating high morbidity and mortality. Over the years, several therapies have been studied in an attempt to reverse or reduce the damage caused by this vascular disorder, including decompressive craniectomy (DC), a surgical technique reserved for cases that evolve with refractory ICH.

**Methods:**

This study seeks to perform a comparative analysis on the effectiveness of decompressive craniectomy using four randomized clinical trials and the results found in the retrospective study conducted in a neurosurgical reference center between 2010 and 2018.

**Results:**

The total sample consisted of 263 patients, among which 118 were randomized and 145 were part of the retrospective study. The outcome was analyzed based on the modified Rankin Scale (mRS) for 6 and 12 months. The mean time to perform the DC was 28.4 h in the randomized trials, with the late approach (> 24 h) associated with unfavorable outcomes (mRS between 4 and 6).

**Conclusion:**

Compared to the aforementioned studies, the study by Bem Junior et al. shows that a surgical approach in < 12 h had a better outcome, with 70% of the patients treated early classified as mRS 2 and 3 at the end of 12 months (1). Decompressive craniectomy is currently the most effective measure to control refractory ICH in cases of malignant ischemic stroke, and the most appropriate approach before surgery is essential for a better prognosis for patients.

## Introduction

The WHO defines ischemic stroke as the “sudden onset of deficient neurological symptoms” attributable to a brain disorder caused by a circulatory disorder lasting longer than 24 h (1). When the ischemic event is associated with brain edema and refractory intracranial hypertension, affecting 2/3 of the middle cerebral artery area, the cerebrovascular accident (CVA) is characterized as malign ischemic stroke and the prognosis is usually poor despite maximal intensive care treatment (2, 3). Several medical therapies have been proposed to reduce development of brain edema and intracranial pressure, such as hyperventilation and osmotic therapy (4–6). Although, several reports suggest that these therapies may be ineffective and lack evidence of efficacy (4, 5).

As the only therapy, surgical decompression has been proposed for patients with space-occupying hemispheric infarction, seeking to relieve the high intracranial pressure (2, 7–9). Therefore, the therapy seeks to create a compensatory space to accommodate the brain and normalize intracranial pressure, reverting brain tissue shifts (2). Randomized double-blind studies describe their results, with an emphasis on the effectiveness of the decompression procedure, but the functional outcome is questionable. In this study, the authors compare the results of retrospective studies carried out in Brazil, and four randomized clinical trials, in search for which is the best surgical time to proceed to DC and this implication in patients outcomes, based on the modified Rankin Scale (mRS).

## Methods

This is a comparative analysis of results obtained from a retrospective study of patients undergoing decompressive craniectomy to control intracranial hypertension secondary to malignant ischemic stroke, developed at Hospital da Restauração (HR), between March 2010 and March 2018 by Bem Junior et al. and secondary data was taken from four randomized clinical trials, published on the European continent between 2006 and 2011 (10). The main objective of this research is to compare the results of Bem Junior et al. (10) and four randomized clinical trials since these trials suggest that the best time to perform decompressive craniectomy is between 12 and 24 h, and the results from previous studies indicate that the earlier approach can achieve more favorable outcomes (10).

### Retrospective study

Data from the study by Bem Junior et al. was collected from the medical records of the Neurosurgery service of Hospital da Restauração (HR), through a semi-structured collection instrument, which evaluated the following variables: age, sex, comorbidities, time from onset of symptoms to arrival at the hospital, time from symptom onset to neurosurgical procedure, laterality of the ischemic event, preoperative Glasgow Coma Scale (GCS), surgery time, intraoperative complications, postoperative complications, length of hospital stay and clinical/functional status after the procedure, evaluated by the modified Rankin Scale and Glasgow Outcome Scale (10).

The results obtained in the first study were descriptively analyzed using absolute and percentage frequencies, by way of Pearson's chi-square test or Fisher's exact test. The strength of association was analyzed based on the Odds Ratio (OD) and the Confidence Interval (CI). The level of significance in the decision of the statistical tests was 5%, with a confidence interval of 95%. The data was computed using an Excel spreadsheet and the program used to obtain the statistical calculations was IMB SPSS version 25.

### Secondary data

The secondary data used in this comparative analysis was taken from four randomized, double-blind clinical trials, and the choice of these trials was guided by the level of the scientific relevance of the articles, as well as by the number of citations of them in the main studies on the subject. The four selected studies were: DESTINY published in 2007, DECIMAL published in 2007, HAMLET published in 2009, DESTINY II published in 2011 (4, 5, 7, 11).

### Comparative analysis

The analysis and comparison performed in this article were based on the interpretation of data available in the four clinical trials and the retrospective study developed by the group of Bem Junior et al. (10). The variables analyzed and compared were: the age of patients, time for indication and performance of decompressive craniectomy and clinical outcome, using the modified Ranking Scale (mRS) for the latter.

## Results

This comparative analysis was performed with a sample of 263 patients, among which 118 were randomized and 145 were part of the retrospective study.

The clinical characteristics of the 145 patients in the retrospective study are detailed in Table 1. The majority (60%) of the cases were aged between 40 and 64 years, followed by 24.8% who were 65 years of age or older. All cases had an admission National Institutes of Health Stroke Scale (NIHSS) above 15. The time to perform decompressive craniectomy, from the moment of arrival to admission to the operating room, was above 24 h in 68.3%, between 12 and 24 h in 13.8% and < 12 h in 17.9% of the samples.

**Table 1:** Clinical variables of group studied by Bem Junior et al. (10).

**Variable**	***n* (%)**
Total studied	145
**Age (years)**	
Up to 17	6 (4.1)
18–39	16 (11.0)
40–64	87 (60.0)
65 or more	36 (24.8)
**Associated clinical conditions**	
Yes	130 (89.7)
No	15 (10.3)
**Surgical hours (h)**	
<12	26 (17.9)
12 a 24	20 (13.8)
> 24	99 (68.3)

The outcome was analyzed based on the modified Rankin Scale for 6 and 12 months and is detailed in Tables 2, 3, according to the clinical data considered for this group of patients. During this study, 28 patients died early and weren't included in the outcomes analysis at 6 and 12 months respectively.

**Table 2:** Rankin scale evaluation modified at 6 months, shown by the clinical data of Bem Junior et al. (10).

	**Rankin Scale (6 months)**				
**Variable**	**4 and 5**	**2 and 3**	**Total**	**OR (CI 95%)**	***P*-value**
***n* (%)**	***n* (%)**	***n* (%)**			
Total group	56 (47.9)	61 (52.1)	117 (100.0)		
**Associated clinical conditions**					*P* ^(1)^ = 0.060
Yes	46 (44.7)	57 (55.3)	103 (100.0)	1	
No	10 (71.4)	4 (28.6)	14 (100.0)	3,1 (0,9 a 10,5)	
**Surgical hours (h)**					
<12	10 (43.5)	13 (56.5)	23 (100.0)	^**^	*P* ^(1)^ < 0.001^*^
12 a 24	1 (5.3)	18 (94.7)	19 (100.0)	^**^	
> 24	45 (60.0)	30 (40.0)	75 (100.0)	^**^	

* Association significant at 5%,

** Weren't calculated due to low frequency.

(1) Test by Qui-square of Pearson.

^(2)^ By Fisher exact test.

**Table 3:** Rankin scale evaluation modified at 12 months, shown by clinical data of Bem Junior et al. (10).

	**Rankin Scale (12 months)**				
**Variable**	**4 and 5**	**2 and 3**	**Total**	**OR (CI 95%)**	***P*-value**
***n* (%)**	***n* (%)**	***n* (%)**			
Total group	35 (29.9)	82 (70.1)	117 (100.0)		
**Associated clinical conditions**					*P* ^(2)^ = 0.756
Yes	30 (29.1)	73 (70.9)	103 (100.0)	1	
No	5 (35.7)	9 (64.3)	14 (100.0)	1.3 (0.4 a 4.4)	
**Surgery hours (h)**					*P* ^(1)^ = 0.916
<12	6 (26.1)	17 (73.9)	23 (100.0)	1	
12 a 24	6 (31.6)	13 (68.4)	19 (100.0)	1.31 (0.34 a 5.01)	
> 24	23 (30.7)	52 (69.3)	75 (100.0)	1.25 (0.44 a 3.59)	

The main characteristics of the sample of patients undergoing DC in the randomized studies included in this analysis are shown in Table 4. Only the study by Jüttler et al. (4) exclusively evaluated patients over 61 years of age, with the mean age of cases in the DECIMAL (7), DESTINY (5) and HAMLET (11) studies being 45.5 years.

**Table 4:** Clinical characteristics of the patients included in the random trials.

**Study**	***n***	**Mean age** **(SD, range)**	**Sex** **(% Male)**	**NIHSS at admission - Mean (range)**	**Associated pre existing conditions**	**Surgical time h Mean (SD range)**	**Mortality**
DECIMAL (7)	20	43.5 (±9.7)	45%	22.5 (16–35)	–	20.5 (± 8.3)	25%
DESTINY (5)	17	43.2 (±9.7)	47%	21 (19–26)	–	24.4 (± 6.9)	18%
HAMLET (11)	32	50.0 (± 8·3)	63%	23 (17–34)	–	41 (29–50)	22%
DESTINY II (4)	49	70 (62-82)	25%	20 (15–40)	–	28 (16–50)	43%

The severity of the ischemic event in these patients assessed by the NIHSS had a mean number of 21.6. The average time to perform the DC in this sample was 28.4 h. The clinical outcome, analyzed based on mRS for 6 and 12 months is specified in Table 5.

**Table 5:** Comparison between outcomes presented by random trials based on mRS at 6 and 12 months.

**Study**	**mRS 6 months**			**mRS 12 months**		
	**2–3**	**4–5**	**6**	**2–3**	**4–5**	**6**
DECIMAL (7)	25%	50%	25%	50%	25%	25%
DESTINY (5)	47%	35%	18%	48%	35%	18%
HAMLET (11)	–	–	–	25%	53%	22%
DESTINY II (4)	7%	60%	33%	6%	51%	43%

## Discussion

Decompressive craniectomy, as a treatment, is an alternative that contributes to positive outcomes in patients who suffer strokes in the 2/3 of the middle cerebral artery territory. The technique used in all cases of the previous study for performing the decompressive craniectomy was a front-temporoparietal hemicraniectomy (12–15 cm) with middle fossa decompression and dural opening.

The assessment of the best surgical time for the procedure is essential for a better outcome. Although it does not reverse the stroke effects and the neuronal loss, the decompressive craniectomy reduces persistent loss in territories beyond the middle cerebral artery or even contralateral by reducing intracranial hypertension and cerebral herniation. The craniectomy approach reported by the four clinical trials took an average of 28.4 h. Analysis using the Rankin Scale or mRS shows that, despite the craniectomy, the relatively late approach (over 24 h) was associated with unfavorable outcomes (mRS between 4 and 6).

The trial by Jüttler et al. had a mean time of 28 h to perform a craniectomy after the onset of symptoms, and demonstrated severe disability (mRS 4–5) and death (mRS 6) in 60 and 33% of cases, respectively, within 6 months (4). In the 12-month follow-up, the prognosis was better, but there were still a high number of patients with severe disabilities (51%) or that died (43%). The study by Hofmeijer et al. (11) in which craniectomy was indicated even later (41 h on average), despite having reported a considerably lower death rate (22%) compared to the study by Jüttler et al. still showed severe patient dysfunction (mRS 4–5) in 53% of cases after 12 months (4, 11). In the study by Jüttler et al. the surgical approach, performed between 24 and 25 h, on average, contributed to a better prognosis, since mortality (mRS 6) remained low and constant (18%), in the periods of 6 and 12 months (5). The trial by Vahedi et al. had, on average, a surgical time of around 20–21 h (7). The approach in a shorter time was essential for a good prognosis, as 75% of the patients survived without severe disability (mRs ≤ 4).

Comparatively, as indicated by data from the retrospective study by Bem Junior (2021) the surgical approach with a time between 12 and 24 h was essential for a more favorable outcome (10). 17% of patients have operated within 12 h of symptom onset and >70% of patients had a relatively favorable outcome (mRS 2 and 3) at 12 months. The performance of a decompressive craniectomy between 12 and 24 h, however, seems to be ideal, according to the data of this study. Of all 20 patients treated in this interval, 95% had a favorable outcome (mRS 2 and 3) 6 months after treatment.

Patients who underwent surgery 24 h after the onset of symptoms had a worse outcome than those who underwent surgery between 12 and 24 h. Of the 75 patients in the first group, 30.7% suffered a moderate to severe disability (mRS 4–5), reinforcing the thesis that performing craniectomy between 12 and 24 h or in < 12 h has a better outcome.

Regarding age, performing decompressive craniectomy in patients over 60 years of age is associated with a worse outcome, as shown by data from the clinical trial by Jüttler et al. (4) who only used older patients in their study. Despite this, data from the four clinical trials show that patients of advanced age may have some benefits from the treatment. In the four studies, the mean age of the participants was 52 years, and mortality ranged between 18 and 25%, except for the DESTINY II study, in which the mean age was 70 years and mortality reached 43% at the end of 12 months (4).

In the previous study, the association between the outcome and the age of the patient shows that the younger ones had a better degree of functionality after 12 months (57.7% for those between 40 and 64 years old) (10). Of the elderly over 65 years who underwent the procedure, only 39.3% had a good outcome.

Compared to the studies cited, the study Bem Junior et al. (10) shows that the surgical approach in < 12 h had a better outcome compared to the surgical time of the other trials, which had an average time of between 20.5 and 41 h. The prognosis of patients in this study, through the Rankin scale, shows that patients operated on within < 12 h had a clinically favorable outcome (mRs 2–3) in 55 and 70% of the cases, in the period of 6 and 12 months, respectively (10). While the outcome, although favorable (mRs 2–3), was significantly lower in the other studies, ranging from 7 to 47% in the 6-month period and 6–50% in the 12-month period. Other factors that contribute to a better prognosis of the patients studied by Bem Junior et al. (10), were the length of hospitalization stay, which was fewer in the patients with better clinical outcomes measured by mRS, and the right cerebral hemispheric involvement.

The previous study discussed the importance of the surgical time in decompressive craniectomy, which is rarely discussed in literature, and how the most appropriate approach to the surgical moment is essential for the best prognosis (10). This study not only confirms what the aforementioned trials had already addressed, the choice of decompressive craniectomy as the best treatment for the control of intracranial hypertension secondary to malignant ischemic stroke, but adds that a relatively early approach contributes to a clinically favorable outcome for the patient. At the 6-month follow-up, about 57% of the patients had mRS 2–3 and at the 12-month follow-up, about 74% of the patients also had a grade of 2–3 on the Rankin scale. The individuals in the samples underwent craniectomy at an interval of < 12 h.

## Conclusion

The performance of decompressive craniectomy within 24 h after the onset of symptoms seems to be an effective alternative for the reduction of short and long-term neurological damage in patients diagnosed with malignant stroke in the MCA territory. In the study by Bem Junior et al. (10) 52.1 and 70.1% of patients operated on at 6 and 12 months, respectively, and at 24 h, had a relatively favorable prognosis (mRS 2–3) in comparison to other studies, such as Hamlet, with prolonged approach (more than 40 h) and with a more guarded prognosis (25% of patients with mRS 2–3 at 12 months) (10). New studies indicate that performing the procedure within 12 h after the onset of symptoms may be associated with even better results. In addition, the age group that benefits most from the procedure is 65 years or under. Bem Junior et al. (10) corroborate that the early adoption of craniectomy is better since the prognostic factors in the postoperative outcomes are favorable. Further studies on the topic are needed to better assess these variables.

## Research limitations

We are aware of the flaws, the study has limitations. We are aware of the selection bias between studies, as the study is a retrospective cohort produced in another country and at different time, and the other studies are prospective. The central proposal of the study is to evaluate and propose what could justify a better outcome, based on early intervention. Furthermore, the anticoagulation use was not analyzed in this research and the cranioplasty was not performed due to service demand.

## Author contributions

Conceptualization, methodology, validation, formal analysis, investigation, resources, writing—original draft, project administration, and supervision: LB. Data curation, writing—original draft, investigation, formal analysis, resource, and writing—review and editing: AS, MM, MG, JA, and OF. Conceptualization, validation, formal analysis, resources, project administration, and supervision: NR, NA, MV, and HA. All authors contributed to the article and approved the submitted version.
